# Trend and projection of the prevalence and burden of near vision loss in China and globally from 1990 to 2030: A Bayesian age-period-cohort modeling study

**DOI:** 10.7189/jogh.14.04119

**Published:** 2024-08-02

**Authors:** Yanxin Xu, Yan Mao, Xuming Lin, Zongyin Gao, Xiaoting Ruan

**Affiliations:** 1 Department of Ophthalmology, Guangzhou First People's Hospital, School of Medicine, South China University of Technology, Guangzhou, Guangdong, China; 2 State Key Laboratory of Ophthalmology, Zhongshan Ophthalmic Center, Sun Yat-sen University, Guangzhou, Guangdong, China; 3 Department of Ophthalmology, Yantai Yuhuangding Hospital, Qingdao University, Yantai, Shandong, China

## Abstract

**Background:**

Few studies have investigated near vision loss (NVL) in China. To address this gap, we aimed to explore trends in the prevalence and disease burden of NVL from 1990 to 2019 and to predict trends over the next decade.

**Methods:**

Using data from the Global Burden of Disease 2019 study, we calculated the age-standardised prevalence rate (ASPR), age-specific disability-adjusted life years (DALYs), and annual percentage change (EAPC) in China and different regions. We then used the Bayesian age-period-cohort (BAPC) predictive model to predict the prevalence trends from 2020 to 2030 in both contexts.

**Results:**

At the global level, ASPRs increased from 5613.27 in 1990 to 5937.81 per 100 000 population in 2019, with an EAPC of 0.06. The ASPR in China specifically decreased from 7538.14 in 1990 to 7392.86 per 100 000 population in 2019 (EAPC = −0.02). The age-standardised DALY rate was higher in women than in men, both globally and in China. The NVL burden was relatively higher in low-income regions, low sociodemographic index regions, and the South-East Asia Region compared to other regions. The predictive model indicated that the ASR trend for NVL slowly increased at a global level after 2020, yet decreased in China.

**Conclusions:**

Despite a decline in the age-standardised prevalence of NVL in China over the next decade, the current burden remains substantial. To alleviate this burden, decision-makers should adopt inclusive approaches by involving all stakeholders.

The rapidly rising trends of population ageing in recent decades have led to a significant increase in the prevalence of near vision loss (NVL) [1], defined as the progressive inability to focus on nearby objects with ageing. Uncorrected presbyopia is the most common cause of NVL in middle-aged and elderly populations [2], with estimates suggesting that 1.8 billion individuals worldwide are affected by this condition, 826 million of whom have NVL [3]. In turn, the impact of NVL on quality of life can be as substantial as that caused by distance vision impairment, regardless of an individual’s environment, lifestyle, or sociodemographic status [4]. People with vision impairment experience lower levels of educational achievement, lower employment rates, higher rates of depression and anxiety, and a higher risk of falls and fractures than those without it [5–7]. Thus, NVL also imposes a substantial economic burden on society [8].

With global population growth and ageing, the number of individuals affected by NVL will likely also increase in the coming years [3]. China ranks among the nations with the highest prevalence of visual impairment, bearing a substantial population of affected individuals globally [9].

The Global Burden of Disease (GBD) study offers a comprehensive overview of mortality and disability worldwide while considering factors such as country, time, age, and sex. Specifically, it quantifies the effect of 369 diseases, injuries, and health-related risk factors in 204 countries and territories, enabling decision-makers to improve healthcare systems within their context and reduce disparities between subgroups [10]. The GBD study monitors several primary causes of blindness and vision impairment, including cataracts, age-related macular degeneration, glaucoma, refractive disorders, and NVL, with the data on NVL helping direct efforts toward prevention and treatment, particularly in China [10]. Despite this, few studies have explored the burden and trends of NVL in the Chinese population.

With this in mind, we collected the prevalence and disability-adjusted life years (DALYs) of NVL, stratified by age, sex, the sociodemographic index (SDI), and income regions, and further calculated age-standardised prevalence rates (ASPRs) and estimated annual percentage changes (EAPCs). We also predicted the trends in NVL over the next 10 years, aiming to provide preliminary data for informing healthcare policy decisions. Our main data source for this was the GBD 2019 Study, which allowed us to perform analyses both at the global level and in China.

## METHODS

### Data sources

We retrieved data on the prevalence and disease burden of NVL from the Global Health Data Exchange [11], which hosts the GBD 2019 database on the incidence, prevalence, mortality, years of life lost, years lived with disability, and DALYs due to 369 diseases and injuries in 204 countries and territories [12,13]. We otherwise retrieved demographic data from 2020 to 2030 from a publicly accessible website [14], and the GBD world population age-standardised data from previously published research [15]. We generated global maps using the data visualisation tool offered by the Institute for Health Metrics and Evaluation within the Global Health Data Exchange [16].

### Data analysis

We used descriptive analysis to assess the burden of NVL at the regional, national, and global levels. First, we calculated the ASPR and EAPC to show the trends of NVL globally and in China, according to a previously described methodology [17]. Second, we calculated the age-standardised prevalence and DALY rates, stratified by age and sex, to determine the related trends of NVL both globally and in China. DALYs serve as a measure of the overall burden of disease, combining the years of life lost due to premature death and the years lived with a disability. Furthermore, we used disease mapping to visualize the age-standardised prevalence and DALY rates per 100 000 population at the national level. We then compared the ASPR and burden of NVL between China and different World Health Organization (WHO), World Bank income, and SDI regions. Finally, we set up a Bayesian age-period-cohort analysis (BAPC) model with integrated nested Laplace approximation to predict the future trend of NVL prevalence from 2020 to 2030 both globally and in China.

We performed all analyses using GraphPad Prism version 9.5.0 (GraphPad Prism Software, San Diego, CA, USA) or R, version 4.3 (R Core Team, Vienna, Austria). For the BAPC predictive model, we used the ‘BAPC,’ version 0.0.36 and ‘INLA, version 23.04.24 packages in R.

## RESULTS

**Figure 1:**
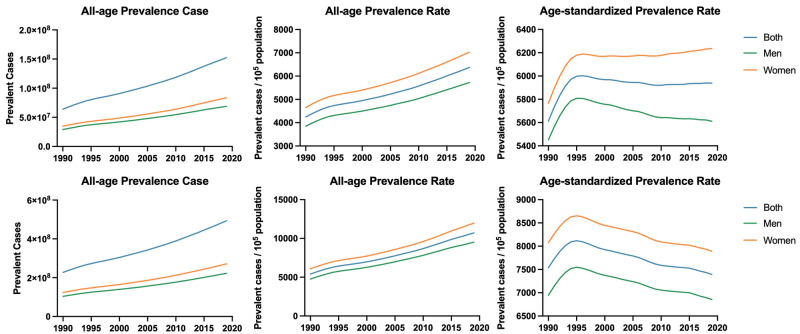
Prevalent cases, prevalence rates, and age-standardised prevalence rates of NVL from 1990 to 2019, globally (**panels A, B, and C**) and in China (**panels D, E, and F**).

Globally, the prevalent cases of NVL increased by 117.09% in our study period, from 227 172.40 **× **10^3^ in 1990 to 493 160.26 **× **10^3^ in 2019; they were also higher among women than men. The unadjusted population prevalence rate increased by 50.10% from 4246.33 per 100 000 population in 1990 to 6373.67 per 100 000 population in 2019, while the ASPR showed much less variation, especially after 1996 (5613.27 per 100 000 population in 1990, 6002.94 per 100 000 population in 1996, and 5937.81 per 100 000 population in 2019). In China, the prevalent cases increased by 138.35% from 63 938.35 **×** 10^3^ in 1990 to 152 396.18 **×** 10^3^ in 2019. Similarly, the unadjusted population prevalence rate also increased by 98.35% over the study period, from 5401.65 per 100 000 population in 1990 to 10 714.39 per 100 000 population in 2019. However, after adjusting for age, the ASPR of 7538.14 per 100 000 population in 1990 peaked in 1995 at 8117.19 per 100 000 population, only to decrease with a prevalence rate of 7392.86 per 100 000 population in 2019 (**Figure 1**, **Table 1**).

**Table 1:** The prevalent cases and ASPRs of NVL in 1990 and 2019, and their temporal trends from 1990 to 2019

	1990		2019		1990–2019
**Characteristic**	**Number of prevalent cases × 10^3^ (95% UI)**	**ASPR per 100 000 population (95% UI)**	**Number of prevalent cases × 10^3^ (95% UI)**	**ASPR per 100 000 population (95% UI)**	**EAPC (95% UI)**
Global	227 172.40 (164 158.06, 298 408.07)	5613.27 (4081.10, 7335.09)	493 160.26 (358 888.66, 645 549.05)	5937.81 (4336.43, 7772.47)	0.06 (0.04, 0.07)
*Men*	103 602.12 (74 847.06, 136 863.09)	5450.91 (3947.80, 7149.57)	222 101.79 (161 222.81, 292 608.00)	5611.61 (4090.65, 7377.45)	0.03 (0.01, 0.05)
*Women*	123 570.12 (89 622.57, 161 772.83)	5766.14 (4191.93, 7523.61)	271 058.47 (198 117.12, 353 362.15)	6235.98 (4558.31, 8128.16)	0.08 (0.06, 0.10)
China	63 938.34 (45 784.11, 84 511.48)	7538.14 (5453.68, 9969.59)	152 396.18 (109 252.73, 200 695.11)	7392.86 (5343.36, 9717.21)	−0.02 (−0.05, 0.01)
*Men*	29 041.57 (20 682.13, 38 873.30)	6946.32 (5008.72, 9237.48)	68 909.16 (49 400, 67, 91 158.51)	6855.83 (4961.84, 9072.61)	−0.01 (−0.04, 0.01)
*Women*	34 896.77 (25 034.37, 46 059.62)	8075.14 (5819.75, 10 640.73)	83 487.02 (59 589.46, 110 098.54)	7890.47 (5698.32, 10 383.64)	−0.02 (−0.06, 0.01)
SDI					
*Low*	26 640.94 (19 914.99, 33 856.88)	10 559.83 (7982.22, 13 325.51)	56 980.87 (42 936.12, 72 263.42)	10 200.10 (7694.59, 12 849.03)	−0.04 (−0.06, −0.02))
*Low-middle*	59 970.45 (42 273.96, 79 381.84)	9527.72 (6918.64, 12 430.87)	126 607.26 (91 649.59, 166 573.19)	8939.80 (6505.52, 11 745.84)	−0.06 (−0.08, −0.06)
*Middle*	71 333.74 (51 299.83, 94 168.19)	6914.85 (5002.69, 9121.16)	170 352.99 (122 324.60, 224 327.90)	6698.88 (4854.51, 8873.46)	−0.03 (−0.05, −0.01)
*High-middle*	57 905.06 (41 536.24, 76 598.64)	5440.67 (3942.79, 7200.87)	117 494.18 (84 969.77, 155 359.28)	5769.35 (4208.80, 7581.53)	0.06 (0.03, 0.09)
*High*	11 235.33 (8020.72, 15080.66)	1097.92 (783.92, 1469.49)	21 548.60 (15 608.12, 28 685.51)	1233.67 (895.74, 1634.11)	0.12 (0.09, 0.17)
World Bank income levels					
*High income*	14 032.39 (9897.29, 18 956.59)	1107.90 (783.40, 1487.37)	230 50.68 (161 73.80, 312 21.22)	1070.86 (758.84, 1445.62)	−0.03 (−0.05, −0.02)
*Upper middle income*	100 949.42 (72 393.03, 133 595.19)	6717.40 (4877.75, 8902.90)	224 883.36 (161 628.28, 296 895.69)	6575.49 (4781.60, 8695.44)	−0.02 (−0.04, 0.00)
*Lower middle income*	95 871.63 (69 748.35, 126 647.47)	8896.68 (6488.81, 11 586.08)	211 963.94 (153 937.64, 277 851.53)	8599.53 (6286.86, 11 217.82)	−0.03 (−0.05, −0.01)
*Low income*	16 231.22 (12 193.24, 20 416.84)	10 382.80 (7820.08, 12 922.85)	33 084.52 (25 187.24, 41 402.86)	9778.65 (7451.73, 12 289.27)	−0.05 (−0.09, −0.03)
WHO region					
*Eastern Mediterranean Region*	9633.76 (7081.59, 12 515.28)	5267.65 (3882.59, 6863.80)	19 672.45 (14 475.42, 25 665.52)	4503.16 (3139.72, 5899.35)	−0.15 (−0.17, −0.12)
*European Region*	35 075.50 (24 991.85, 46 492.11)	3278.34 (2349.19, 4317.41)	49 585.07 (35 448.35, 66 171.13)	3200.90 (2309.12, 4244.67)	−0.02 (−0.04, −0.01)
*Region of the Americas*	13 185.49 (9531.50, 17 638.79)	2168.12 (1564.56, 2895.88)	31 624.30 (22 728.73, 42 140.59)	2497.75 (1802.67, 3334.78)	0.15 (0.13, 0.18)
*South-East Asia Region*	75 317.50 (54 761.69, 98 749.08)	10 115.84 (7382.39, 13 155.00)	173 751.81 (126 966.87, 227 299.98)	9675.35 (7103.81, 12664.29)	−0.04 (−0.07, −0.02)
*Western Pacific Region*	70 684.20 (50 584.64, 93 527.47)	5954.68 (4284.51, 7868.33)	167 193.40 (119 497.77, 220 487.21)	6042.60 (4361.05, 7942.52)	0.01 (−0.01, 0.05)
*African Region*	22 577.46 (16 615.31, 28 985.42)	9695.78 (7178.29, 12 391.46)	49 760.46 (37 062.30, 63 839.51)	9406.49 (6992.88, 12 012.88)	−0.03 (−0.05, −0.01)

ASPR – age-standardised prevalence rate, EAPC – estimated annual percentage change, UI – uncertainty interval

**Figure 2:**
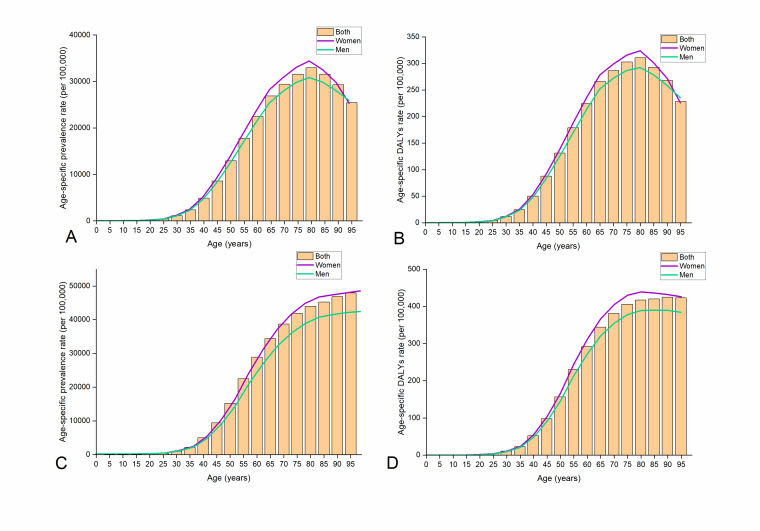
Age-specific prevalence rate and DALYs rate of NVL by age and gender in 2019, globally (**panels A and B**) and in China (**panels c and D**). DALY – disability-adjusted life year.

Moreover, the ASPRs of NVL in 2019 were higher in women than in men, both globally and in China. They also increased with age in both contexts, with the 80–84-year-old age group being most affected globally and the 40–95-year-old age group being most affected in China. Similar to the findings for ASPRs, we found a higher disease burden in women than in men, and in older populations overall. At the global level in 2019, the ASRs of NVL were 61.66 (95% uncertainty interval, UI = 28.31, 121.50) and 55.97 (95% UI = 25.74, 111.18) for women and men, respectively; in China, the ASRs were 78.46 (95% UI = 36.10, 154.07) for women and 68.93 (95% UI = 31.64, 136.12) for men (**Figure 2**).

**Figure 3:**
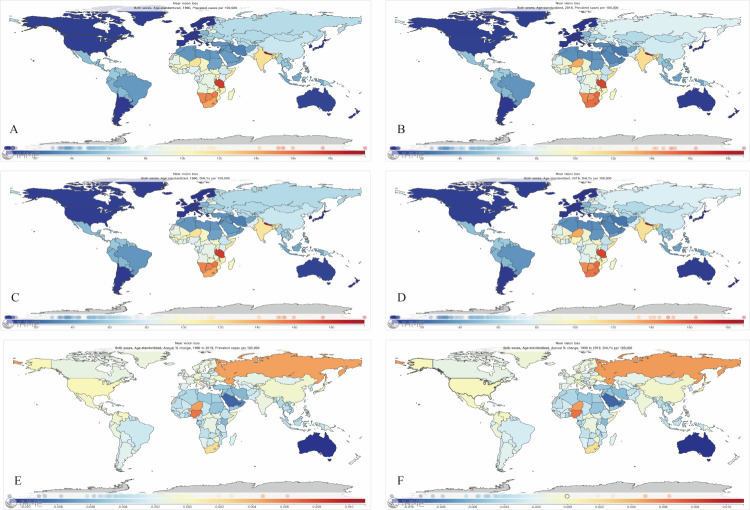
Global map of the age-standardised prevalence rate of NVL in 1990 (**panel A**) and 2019 (**panel B**); age-standardised DALYs rate in 1990 (**panel C**) and 2019 (**panel D**); annual percentage change in the age-standardised prevalence rate (**panel E**); and DALY rate (**panel F**) from 1990 to 2019.

The ASPR per 100 000 population in 1990 was the highest in Nepal (19 894.10; 95% UI = 15 759.29, 23 667.75) and the lowest in Sweden (443.01; 95% UI = 295.49, 623.78). The ASPR per 100 000 population in 2019 was also the highest in Nepal (18 819.53; 95% UI = 15 245.96, 22 104.12) and the lowest in Sweden (427.02; 95% UI = 288.65, 604.69). Similarly, Nepal and Sweden had the highest and lowest age-specific DALY rates, respectively, in both 1990 and 2019. From 1990 to 2019, the annual change of the ASPR was not significant globally. At the national level, however, we found the highest increase in Nigeria (EAPC = 0.62 per 100 0000 population) and the highest decrease in Australia (EAPC = −1.1 per 100 0000 population). We found similar trends for the annual change of DALYs (**Figure 3**).

**Figure 4:**
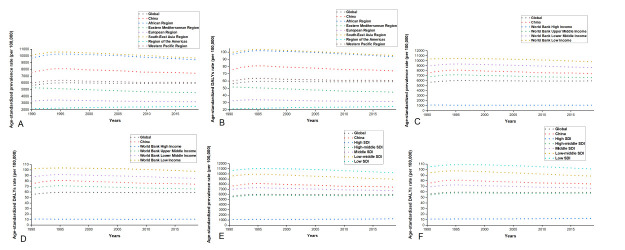
Age-standardised prevalence rate and burden of NVL in 1990–2019 by WHO regions (**panels A** and (**B**); levels of income regions (**panels C** and (**D**); and levels of SDI regions (**panels E** and (**F**). SDI – sociodemographic index.

Among the six WHO regions, the South-East Asia Region had the highest ASPRs and age-standardised DALYs rate between 1990 and 2019, while the Region of the Americas had the lowest values. China ranked third in terms of the ASR and NVL burden. In terms of World Bank income, low-income regions had the highest prevalence rates (9778.65 per 100 000 population), while high-income regions had the lowest (1070.86 per 100 000 population). Additionally, high-income regions tended to suffer from a lower burden of NVL compared to others. Similarly, low-SDI regions had the highest prevalence rates (10 200.10 per 100 000 population), while high-SDI regions had the lowest (1233.67 per 100 000 population). We also observed a much lower disease burden of NVL in high-SDI regions than elsewhere (**Figure 4**).

**Figure 5:**
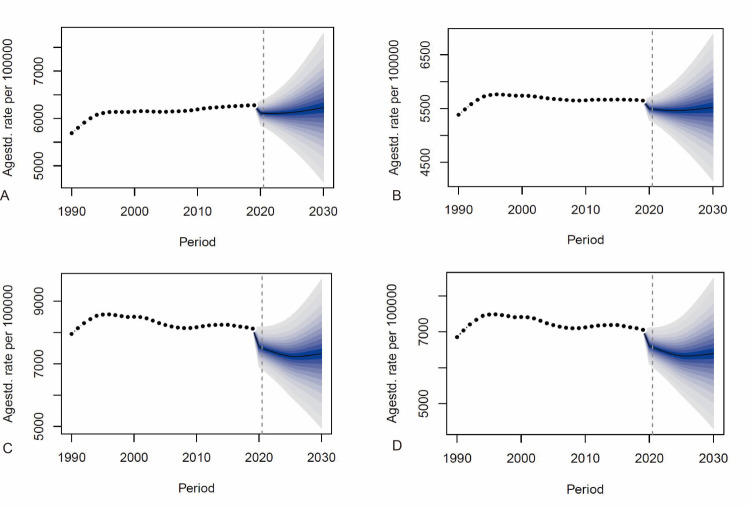
The change in trends of the NVL-related disease burden by sex from 1990 to 2030, globally (in women (**panel A**) and men (**panel B**)) and in China (in women (**panel C**) and men (**panel D**)).

In terms of overall trends, we observed a rapid upward trend until 1996, when it peaked and began to decrease slowly for men and increase slowly for women globally. Our BAPC model predicted a gradual increase in this trend from 2020 onwards, with similar trends for both sexes. In contrast, the ASPR for both sexes in China decreased after 1995, with the predictive modelling suggesting that by 2030, the ASPRs of NVL will decrease to 6606.78 × 10^6^ for men and 7646.10 × 10^6^ for women, with an overall decreasing trend (**Figure 5**).

## DISCUSSION

Here we report on the prevalence trends and disease burden of NVL, both at the global level and in China. From 1990 to 2019, the prevalent cases of NVL increased by 117.09% globally and by 138.35% in China. They initially showed a gradual upward trend in both contexts; after adjusting for population growth and ageing, the ASPRs did not change significantly after 1996 globally, while they showed a fluctuating downward trend in China. The disease burden of NVL showed the same tendency and was higher in the elderly population. In terms of sex, the age-specific prevalence and DALY rates were higher in women than in men. Moreover, the NVL burden was significantly higher in the low-income and low-SDI region and the WHO-defined South-East Asia region than in other comparable contexts. According to our projection models, the global ASPRs of NVL will slowly increase over the next decade, while the trend will decline slowly in China by 2030; however, the disease burden will remain high globally.

The definition of NVL in the GBD 2019 study aligns closely with that of functional presbyopia, which relates to NVL caused by presbyopia that can be corrected optically [18,19]. Understanding and tackling this substantial, yet correctable burden of NVL could help decision-makers design policies and interventions that would improve both individuals’ lives and socioeconomic development in general. Yet despite the WHO’s call for population-based NVL studies in 2015 [3,19], as well as the findings from the GBD 2017 that NVL was the primary cause of blindness and visual impairment [12], there is little research on the epidemiology of NVL or potentially effective interventions [2,20–22].

We found that the prevalent cases of NVL increased by 138.35% in China in our study period, from 63 938.35 **×** 10^3^ in 1990 to 152 396.18 **×** 10^3^ in 2019. This almost twofold increase in prevalence could be largely attributed to the country’s growing and ageing population. Wang et al. [23] reported that the global burden of NVL increased by 82.4% from 1990 to 2017, which is lower than the increase of 138.35% we observed at the global level here. However, after adjusting for age, we found that the ASR has been declining since it peaked in 1995. The reason for this trend is unclear and requires further investigation. Despite this slight decrease in the ASPR of NVL, its burden remains high. At the global level, the prevalence of NVL increased by 50.10% from 1990 to 2019, while the ASPR also showed an increasing trend.

Jin et al. [24] found that the burden of NVL mainly affected the 45–80-year-old age group. We similarly found that the ASPR increased with age both in China and globally, with a higher burden in the older population. We also found that the prevalence of NVL was higher in women than in men; while this is consistent with the findings of previous studies [24], they have also observed that sex does not play a significant role in the prevalence or incidence of NVL [19,25]. Our findings might be attributed to the longer life expectancy and a higher proportion of women among the elderly population. Therefore, future efforts to enhance eye care services should target middle-aged and elderly individuals, as well as women in general.

We also saw clear regional inequality, with a higher disease burden in the WHO-defined South-East Asia and African regions, and higher DALY rates in low-income and low-SDI regions. The burden we observed in China lies between the one we found for upper-middle income and lower-middle income regions and the middle and low-middle SDI regions, which may be attributed to differences in economic levels. This increased burden of NVL in underdeveloped countries and regions might be primarily attributed to limited awareness of and access to eye care, inadequate numbers of facilities and eye care professionals, and lower coverage of spectacles [23,26,27]. Zebardast et al. [28] also found that having a below-high school level of education, lacking private health insurance, and having an income less than the poverty level were associated with high odds of NVL. Another study found that more than 500 million individuals with presbyopia globally could not afford spectacles which were necessary to correct their vision, most of whom resided in low-income and middle-income countries [29]. Previous studies have indicated that the coverage of presbyopia correction spectacles in rural China (51.5%) was significantly lower than that in urban China (87.7%) [19,30]. One study suggested that approximately half of those aged ≥35 years developed NVL; most, however, could correct the condition with spectacles [25]. Spectacles continue to be the most cost-effective solution for correcting NVL and could be further enhanced by improved medical knowledge and public awareness [31].

Our BAPC predictive model showed a slow upward trend in the global prevalence after 2020 and a downward one in China, with consistent trends in both sexes. This may be attributed to the WHO and the International Agency for the Prevention of Blindness, which campaigns for World Sight Day and other lead initiatives, such as 2030 In Sight, the United Nations Friends of Vision group, VISION 2020, and Vision for the Commonwealth [32]. China has also contributed to the prevention of global blindness; since 1996, it adopted the National Eye Care Day initiative, through which it advocates for the importance of eye health and promotes eye care awareness among the general public. All these strategies have improved populations’ knowledge and awareness of eye health, as well as the use and uptake of spectacles and cataract surgery.

This study had some limitations. First, our data came from the GBD database, which has shortcomings such as data availability over time and statistical assumptions, as well as the lack of high-quality data for certain countries where epidemiological studies had not been conducted. It also lacks information on risk factors for NVL, which is why we did not include them in our analysis. Additionally, our prediction model was based on NVL estimates from 1990 to 2019 and therefore may involve both bias and uncertainty, which is why our results should be interpreted with caution. Future studies should attempt to develop more precise and inclusive predictive models.

## CONCLUSIONS

We observed that the disease burden and ASR of NVL have increased globally from 1990 to 2019, while these trends have declined in China. Our predictive model suggests that the ASR will remain low in the next decade; yet due to China’s population growth and ageing, its burden of NVL remains very high. We further found that older populations and women bore a higher burden of NVL. Hence, with the increase in the ageing population, China should pay more attention to NVL-induced low vision and blindness and formulating public policies aimed at mitigating the burden of this disease.
